# Bridging the gaps between randomized controlled trials and real-world use of thrombopoietin receptor agonists for adult primary immune thrombocytopenia: a systematic review and meta-analysis

**DOI:** 10.3389/fmed.2025.1667457

**Published:** 2025-09-24

**Authors:** Liping Luo, Shanshan Jin, Zhujin Song, Gaowei Chong, Haiying Ding, Su Zeng, Mengfei Dai, Miaolian Wu

**Affiliations:** ^1^Department of Pharmacy, The Fourth Affiliated Hospital of School of Medicine, and International School of Medicine, International Institutes of Medicine, Zhejiang University, Yiwu, Zhejiang, China; ^2^College of Pharmaceutical Sciences, Zhejiang University, Hangzhou, Zhejiang, China; ^3^Department of Pharmacy, Zhejiang Hospital, Hangzhou, Zhejiang, China; ^4^Department of Pharmacy, Zhejiang Cancer Hospital, Hangzhou Institute of Medicine (HIM), Chinese Academy of Sciences, Hangzhou, Zhejiang, China; ^5^Research Center for Clinical Pharmacy, College of Pharmaceutical Sciences, Zhejiang University, Hangzhou, Zhejiang, China

**Keywords:** immune thrombocytopenia, meta-analysis, randomized controlled trials, real-world evidence, thrombopoietin receptor agonists

## Abstract

**Background:**

Randomized controlled trials (RCTs) evaluate short-term efficacy/safety of thrombopoietin receptor agonists (TPO-RAs) in immune thrombocytopenia (ITP), leaving long-term outcomes unclear. This study integrates real-world evidence (RWE) with RCT to assess TPO-RA performance across treatment durations.

**Methods:**

A systematic literature search identified RCTs and real-world studies (RWS) assessing TPO-RAs in adults with primary ITP. Short-term (≤6 months) and long-term (6–12/>12 months) outcomes included platelet response, rescue therapy, bleeding events, and adverse events (AEs). Pooled odds ratios (ORs) with 95% confidence intervals (CIs) were calculated using random/fixed-effects models.

**Results:**

Meta-analysis included 12 RCTs and 32 RWS. Short-term TPO-RA administration yielded 70% platelet response versus placebo (OR = 18.07, 95% CI:12.4–26.16, *p* < 0.001), escalating to 85% (6–12 months) and 91% (>12 months) in RWS. TPO-RAs reduced bleeding risks (any: OR = 0.43, significant: OR = 0.40, both *p* < 0.001). Rescue therapy increased from 12% (short-term) to 32% (>12 months). Serious AE (SAE) incidence matched placebo short-term (OR = 0.69, 95% CI:0.47–1.01) but rose from 8% (RCTs) to 27% (RWS > 12 months).

**Conclusion:**

TPO-RAs sustain durable platelet response but exhibit increase in rescue therapy and SAEs over time. Longitudinal RWS integration into ITP management is critical, necessitating protocolized safety monitoring and personalized regiments to optimize chronic TPO-RA utilization.

**Systematic review registration:**

http://www.crd.york.ac.uk/PROSPERO, identifier [CRD42025649608].

## 1 Introduction

Primary immune thrombocytopenia (ITP), an immune-mediated bleeding disorder marked by accelerated platelet destruction and impaired thrombopoiesis, manifests clinically as thrombocytopenia (platelet count <100 × 10^9^/L) with consequent bleeding susceptibility (1). Studies conducted in Western countries report an annual incidence of 2–10 per 100,000 individuals (2, 3). Complete platelet normalization remains an elusive clinical endpoint (4). Current therapeutic interventions prioritize achieving hemostatic platelet thresholds (>50 × 10^9^/L) to prevent severe bleeding, particularly below 30 × 10^9^/L (5, 6). First-line management employs glucocorticoids and intravenous immunoglobulin for rapid platelet elevation, whereas second-line strategies encompass thrombopoiesis-stimulating agents, rituximab, and splenectomy (6). However, there are still many challenges. Despite initial glucocorticoid efficacy in 70–80% of newly diagnosed patients, prolonged exposure heightens risks of metabolic complications (steroid-induced diabetes, osteoporosis), avascular necrosis, and thromboembolic events (7). Splenectomy, though achieving durable remission in ≈66% of cases (4), confers increased susceptibility to sepsis and venous thromboembolism (8). Recombinant human thrombopoietin (rh-TPO) demonstrates limited clinical utility due to suboptimal response rates and relapse propensity (9). Thrombopoietin receptor agonists (TPO-RAs: eltrombopag, avatrombopag, hetrombopag, romiplostim) emerge as promising alternatives, offering enhanced safety profiles and sustained efficacy compared to conventional therapies (4, 10), and TPO-RAs have also been found to be safe and effective in the treatment of ITP in pediatric patients (11). Furthermore, TPO-RAs have also demonstrated a positive impact on health-related quality of life (HRQoL) (12). By effectively reducing the risk of bleeding episodes and the dependency on rescue therapies, TPO-RAs therapy can significantly enhance patients’ physical function. Consequently, TPO-RAs is an essential consideration in the long-term management strategy for ITP.

Randomized controlled trials (RCTs) have validated the capacity of TPO-RAs to elevate platelet counts to hemostatic thresholds (≥50 × 10^9^/L), reduce hemorrhage incidence, and maintain favorable tolerability profiles within short-term therapeutic windows (≤6 months) (13, 14). However, the constrained observational frameworks of RCTs—typically limited to ≤6-month follow-up durations—restrict robust evaluation of longitudinal efficacy and safety outcomes. This methodological limitation underscores the complementary role of real-world studies (RWS) (i.e., non-interventional, observational studies such as prospective and retrospective cohort studies), which employ extended surveillance periods (>6 months) to assess sustained therapeutic performance (15, 16).

To bridge the gaps between the short-term efficacy and safety data from RCTs and the need for long-term evidence in clinical practice, this meta-analysis introduces a novel approach by comprehensively synthesizing data from both RCTs and RWS. Unlike previous meta-analyses that were exclusively based on short-term RCT data (typically ≤6 months), this research uniquely integrates long-term evidence derived from RWS. This methodology not only enhances the external validity of the findings but also provides pivotal insights into the sustained effectiveness and safety of TPO-RAs in the management of ITP, thereby generating more actionable and clinically relevant evidence to guide long-term therapeutic decision-making.

## 2 Methods

### 2.1 Search strategy and selection criteria

This investigation was prospectively registered in PROSPERO (CRD42025649608) and rigorously adhered to the PRISMA (Preferred Reporting Items for Systematic Reviews and Meta-analyses) guidelines (17). A systematic literature search was executed across four major biomedical databases (PubMed, Embase, Web of Science, Cochrane Library) encompassing all available records from database inception to January 23, 2025, without linguistic exclusion criteria. The search framework incorporated three principal domains: (1) patient population (immune thrombocytopenia diagnosis), (2) therapeutic interventions (thrombopoietin receptor agonists: romiplostim, eltrombopag, avatrombopag, hetrombopag), and (3) study designs (randomized controlled trials, prospective/retrospective observational studies, cohort studies, case–control studies). Boolean operators combined MeSH terms and free-text keywords specific to each conceptual domain (complete strategy detailed in Supplementary Table S1). To ensure comprehensive coverage, citation tracking and manual bibliography reviews supplemented electronic searches.

Inclusion criteria encompassed: (i) adults with a primary ITP diagnosis; (ii) therapeutic regimens involving TPO-RAs; (iii) placebo-controlled randomized trials or observational studies (prospective or retrospective cohort studies); (iv) documented efficacy and/or safety outcomes. Conversely, exclusion parameters comprised: (i) secondary ITP or pediatric populations; (ii) studies lacking TPO-RA treatment duration specifications; and (iii) publication types considered non-primary research or insufficient for robust evidence synthesis, including reviews, editorials, conference abstracts, case reports and case series, and preclinical models, which cannot provide direct and quantitative evidence on drug effectiveness and safety. In instances where multiple publications originated from the same RCT, the publication containing maximal endpoint granularity was prioritized. Two investigators (LP Luo and MF Dai) independently performed literature screening, with arbitration by a third researcher (ZJ Song) to resolve selection discrepancies.

### 2.2 Data extraction, outcomes and quality assessment

Two researchers independently executed dual extraction of critical variables through standardized data collection form, capturing: (i) study metadata (authorship, publication year, design methodology, sample size, therapeutic protocols); (ii) demographic-clinical parameters (age distribution, disease stage, baseline platelet levels, splenectomy rates, TPO-RA exposure duration); (iii) key elements of risk of bias assessment; and (iv) granular outcome parameters encompassing efficacy metrics and adverse event profiles.

The efficacy and safety outcomes included the following (18): (1) Overall platelet response: operationalized as achieving ≥50 × 10^9^/L platelets during treatment; (2) Clinically relevant response: defined by dual thresholds—absolute platelet count ≥30 × 10^9^/L with ≥100% increase from baseline; (3) Durable platelet response: maintenance of platelet levels ≥50 × 10^9^/L for ≥75% of treatment duration; (4) Rescue therapy: defined as the use of any medication aimed at increasing platelet counts or preventing bleeding; (5) Any bleeding: graded WHO Bleeding Scale (Grade 1–4); (6) Significant bleeding: restricted to WHO Grades 2–4 manifestations (19); (7) Any adverse event: encompassing all CTCAE-classified events; (8) Serious adverse event (SAE): events meeting CTCAE v5.0 Grade 3–5 criteria (20).

Methodological rigor was assessed using the Cochrane Risk of Bias Tool for RCTs, evaluating six core domains: random sequence generation, allocation concealment, blinding of participants and personnel, incomplete outcome data, selective outcome reporting, and other biases (21). Extension studies originating from parent RCTs inherited their progenitor trials’ bias assessments. Non-randomized single-arm investigations underwent quality evaluation via the Joanna Briggs Institute (JBI) Case Series Appraisal Tool, a standardized instrument examining 10 methodological criteria encompassing case selection criteria, evaluation of the disease or health condition, and the presentation of case data. Inter-rater discrepancies in quality assessments were resolved through iterative peer deliberation with an independent methodologist (ZJ Song), ensuring consensus-based adjudication.

### 2.3 Statistical analysis

For RCTs, efficacy and safety outcomes were analyzed via odds ratios (ORs) with 95% confidence intervals (CIs), employing either fixed-effects or random-effects models contingent on interstudy heterogeneity levels (22). Heterogeneity thresholds (*I*^2^ > 50% or *p* < 0.05) dictated model selection, with the fixed-effects approach reserved for homogeneous datasets (*I*^2^ ≤ 50%) and random-effects models applied to heterogeneous cohorts (22). In contrast, RWE-derived single-arm investigations were analyzed through pooled event rates with 95% CIs, utilizing random-effects meta-analyses by default to accommodate inherent variability across observational study designs.

Longitudinal subgroup stratification was conducted according to therapeutic exposure periods (<6 months, 6–12 months, >12 months), stratified by median/mean TPO-RA treatment durations reported in source studies. This stratification framework aimed to delineate temporal patterns in therapeutic performance, particularly addressing pharmacodynamic sustainability and cumulative safety profiles in prolonged pharmacotherapeutic regimens. The potential publication bias of RCTs was assessed through funnel plots. All meta-analyses were executed using STATA 15.1 (Stata, College Station, TX, United States).

## 3 Results

### 3.1 Search results and characteristics of studies included

**Figure 1:**
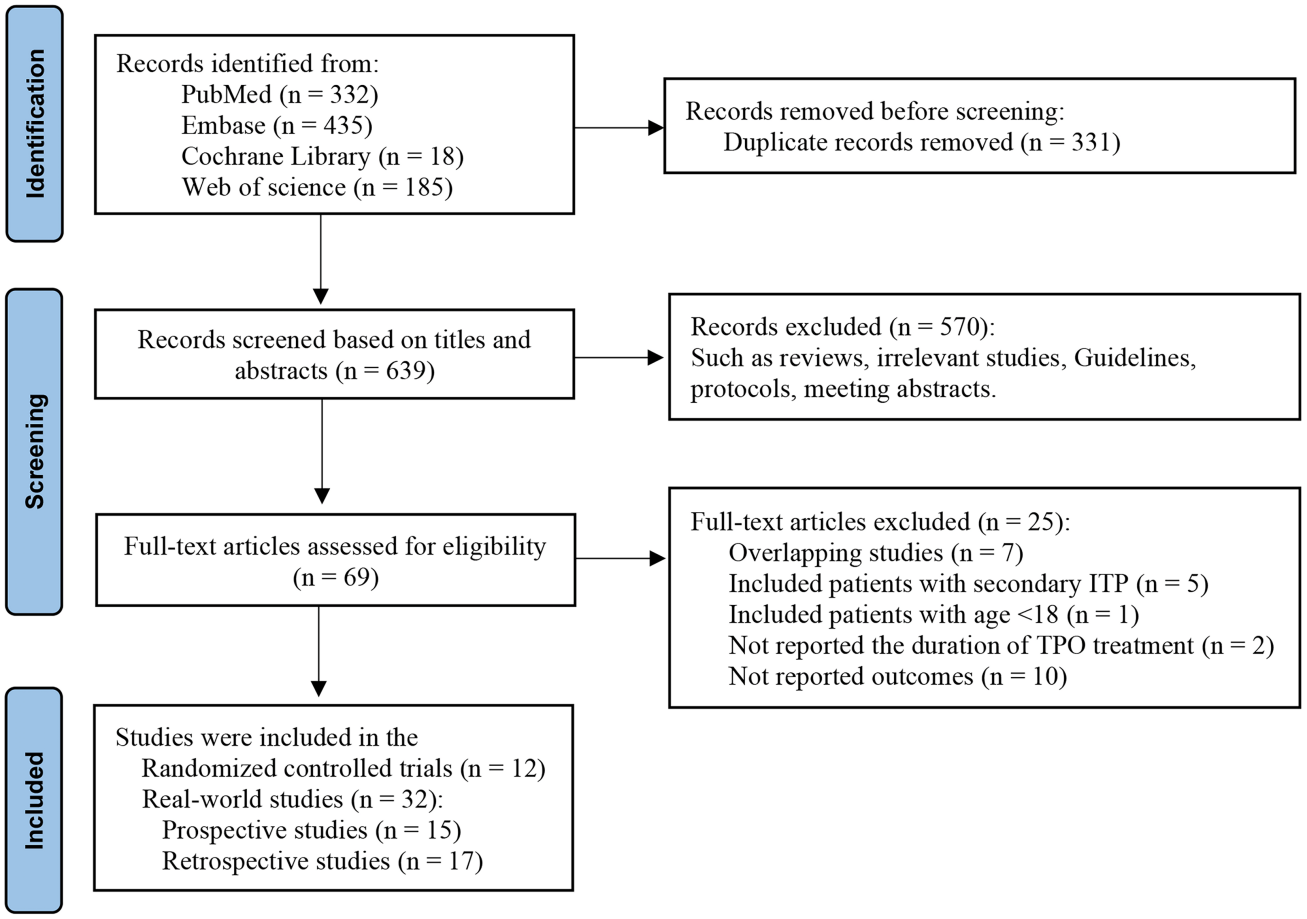
Flow diagram for selection of eligible studies.

The systematic literature search yielded 970 initial records from electronic databases, with 331 duplicates removed through automated deduplication protocols. Following title/abstract screening using predefined eligibility criteria, 570 non-conforming records were excluded. Full-text evaluation of the remaining 69 publications resulted in 44 studies meeting inclusion criteria for final analysis (Figure 1). The analysis included 12 RCTs (12–15, 23–30) and 32 RWS [15 prospective studies (14–16, 24, 26, 28, 31–39), 17 retrospective studies (40–56), all are single-arm cohort studies]. This consistent single-arm design reflects the ethical and subject protection imperative in observational research to provide active treatment to all enrolled patients with a confirmed clinical diagnosis, thereby forgoing placebo control groups.

The 12 RCTs collectively enrolled 1,578 adult patients with primary ITP (Table 1). These trials evaluated four TPO-RAs: romiplostim (3 trials, *n* = 361) (13, 27, 30), eltrombopag (5 trials, *n* = 606) (12, 23, 25, 28, 29), avatrombopag (3 trials, *n* = 187) (15, 24, 26) and hetrombopag (1 trial, *n* = 424) (14). All participants met stringent inclusion criteria, including a confirmed ITP diagnosis for ≥3 months, baseline platelet counts < 30 × 10^9^/L, and prior exposure to ≥1 ITP therapy. Treatment durations in the RCTs primarily spanned <6 months, with two exceptions reporting 25- and 26-week protocols. Outcome data were primarily collected within the 6-month period, allowing the RCTs data to reflect the efficacy and safety of TPO-RAs treatment within 6 months.

**Table 1:** The basic characteristics of the included studies (randomized controlled trials).

Author (published year)	Trial ID (phase)	Intervention (*n*)	Control (*n*)	Disease stage	Initial dose	Age (years, I/C)	Splenectomy (*n*/%, I/C)	Duration of treatment
Al-Samkari (2022) (15)	NCT01438840 (III)	Avatrombopag (*n* = 32)	Placebo (*n* = 17)	≥12 months	20 mg/day	Mean 46.4 (14.2)/41.2 (14.7)	11 (34. 4)/5 (29.4)	24 weeks
Bussel (2007) (23)	NCT00102739 (III)	Eltrombopag (*n* = 88)	Placebo (*n* = 29)	≥6 months	30 or 50 or 75 mg/day	Median 50 (18–85)	41 (46.6%)/14 (48%)	6 weeks
Bussel (2009) (25)	NCT00102739 (III)	Eltrombopag (*n* = 76)	Placebo (*n* = 38)	≥6 months	50 mg/day	Median 47 (19–84)/51 (21–79)	31 (41%)/14 (37%)	6 weeks
Bussel (2014) (24)	NCT00441090 (II)	Avatrombopag (*n* = 59)	Placebo (*n* = 5)	≥3 months	2.5 or 5 or 10 or 20 mg/day	Mean 53.6/40	18 (30.5%)/2 (40%)	4 weeks
Cheng (2011) (12)	NCT00370331 (III)	Eltrombopag (*n* = 135)	Placebo (*n* = 62)	≥6 months	50 mg/day	Median 47.0 (34–56)/52.5 (43–63)	50 (37%)/21 (34%)	26 weeks
Kuter (2008) (13)	NCT00102323 and NCT00102336 (III)	Romiplostim (*n* = 83)	Placebo (*n* = 42)	≥12 months	1 μg/kg/week	Median 52 (21–88)	42 (50.6%)/21 (50%)	25 weeks
Mei (2021) (14)	NCT03222843 (III)	Hetrombopag (*n* = 339)	Placebo (*n* = 85)	≥6 months	2.5 or 5 mg/day	Median 40	29 (8.5%)/4 (4.7%)	10 weeks
Mei (2023) (26)	CTR20210431 (III)	Avatrombopag (*n* = 48)	Placebo (*n* = 26)	≥12 months	20 mg/day	Mean 43.4 (15.4)/47.6 (13.1)	2 (4.2%)/4 (15.4%)	6 weeks
Shirasugi (2011) (27)	NCT00603642 (III)	Romiplostim (*n* = 22)	Placebo (*n* = 12)	≥6 months	3 μg/kg/week	Mean 58.5 (12.6)/47.6 (13.4)	10 (45.5%)/5 (41.7%)	12 weeks
Tomiyama (2012) (28)	NCT00540423 (II/III)	Eltrombopag (*n* = 15)	Placebo (*n* = 8)	≥6 months	12.5 mg/day	Median 58.0 (26–72)/60.5 (38–72)	5 (63%)/11 (73%)	6 weeks
Yang (2017) (29)	NCT01762761 (III)	Eltrombopag (*n* = 104)	Placebo (*n* = 51)	≥12 months	25 mg/day	Mean 44.7 (15.9)/41.3 (12.8)	18 (17.3%)/7 (13.7%)	6 weeks
Zhou (2023) (30)	NCT02868099 (III)	Romiplostim (*n* = 151)	Placebo (*n* = 51)	≥6 months	1 μg/kg/week	Mean 42.1 (14.0)/39.7 (13.9)	14 (9.3%)/5 (9.8%)	9 weeks

All patients have a baseline platelet count less than 30 × 10^9^/L of blood, and have received at least one previous treatment for ITP. C, Control; I, Intervention.

The 15 prospective investigations (Table 2) encompassed 2,513 primary ITP adults, comprising single-arm designs with maximum follow-up durations extending to a mean of 110 weeks. Seven studies (14, 15, 24, 26, 28, 33, 36) functioned as RCT extensions evaluating extended TPO-RA regimens, while three investigations (34, 37, 38) incorporated newly diagnosed ITP patients. Nearly all enrolled subjects presented with baseline thrombocytopenia (<30 × 10^9^/L). Seventeen retrospective studies (Table 3) involving 2,238 ITP patients demonstrated heterogeneous designs: 12 multicenter collaborations and 6 investigations including newly diagnosed cases, with therapeutic follow-up durations reaching a median of 25 months. In summary, these RWS provided longitudinal safety/efficacy data exceeding conventional RCT timelines.

**Table 2:** The basic characteristics of the included studies (prospective studies).

Author (published year)	Type of study (trial ID)	Drug (*n*)	Disease stage	Baseline platelet counts (*10^9^/L)	Duration of treatment
Al-Samkari (2022) (15)	Extension study of RCT (NCT01438840)	Avatrombopag (*n* = 47)	Chronic ITP	/	Mean 44 weeks
Bussel (2014) (24)	Extension study of RCT (NCT00441090)	Avatrombopag (*n* = 53)	Chronic ITP	/	28 weeks
Liu (2022) (33)	Extension study of RCT (NCT01762761)	Eltrombopag (*n* = 150)	Chronic ITP	Mean 19.7 (15.4)	30 weeks
Mei (2021) (14)	Extension study of RCT (NCT03222843)	Hetrombopag (*n* = 339)	Chronic ITP	/	24 weeks
Mei (2023) (26)	Extension study of RCT (CTR20210431)	Avatrombopag (*n* = 72)	Chronic ITP	/	26 weeks
Tomiyama (2012) (28)	Extension study of RCT (NCT00540423)	Eltrombopag (*n* = 23)	Chronic ITP	Median 17 (10–24)	30 weeks
Shirasugi (2012) (36)	Extension study of RCT (NCT00603642)	Romiplostim (*n* = 44)	Chronic ITP	Median 16.5 (3, 32)	Mean 102 weeks
Janssens (2015) (31)	Prospective study (NCT00508820)	Romiplostim (*n* = 470)	\	Median 14 (0–170)	Median 44.3 (20.4, 65.9) weeks
Kuter (2013) (32)	Prospective study (NCT00116688)	Romiplostim (*n* = 292)	Chronic ITP	Median 35 (15–100)	Mean 110 weeks
Newland (2016) (35)	Prospective study (NCT01143038)	Romiplostim (*n* = 75)	\	Median 20 (12–25)	Median 51 (0.3–52.4) weeks
Wong (2017) (16)	Prospective study (NCT00351468)	Eltrombopag (*n* = 302)	Chronic and persistent ITP	<30 (70%)	Median 2.37 years
Lucchini (2021) (34)	Prospective study (NCT2402998)	Eltrombopag (*n* = 51)	Newly and persistent ITP	Median 19 (1–277)	24 weeks
Snell Taylor (2021) (37)	Prospective study	Romiplostim (*n* = 340)	Newly, persistent and chronic ITP	Median 20 (0, 380)	Median 24 (1.0, 24) weeks
Tripathi (2014) (38)	Prospective study	Eltrombopag (*n* = 27)	Newly ITP	Mean 17.5 (3.6)	3 months
Wong (2023) (39)	Prospective study	Eltrombopag (*n* = 228)	Chronic ITP	Median 19.0 (1–495)	Median 484.5 (1–642) days

All studies were prospective single-arm studies. ITP, Immune thrombocytopenia; RCT, randomized controlled trials.

**Table 3:** The basic characteristics of the included studies (retrospective studies).

Author (published year)	Study design	Drug (*n*)	Number of prior therapies (*n*)	Disease stage	Baseline platelet count (^*^10^9^/L, Median)	Duration of treatment
Arnall (2021) (40)	Single-center	Romiplostim and Eltrombopag (*n* = 107)	≥1	Relapsed/refractory ITP	Rom: 23 (2–132) Elt: 29 (3–160)	6 months
Çekdemir (2019) (41)	Multi-center	Eltrombopag (*n* = 285)	\	Chronic ITP	\	Mean 18.0 (6.4) months
Cooper (2024) (42)	Multi-center	Eltrombopag and Romiplostim (*n* = 218)	Median 3.0 (2.0–4.0)	Chronic ITP	17.0 (7.2–34.0)	12 weeks
Dong (2024) (43)	Single-center	Eltrombopag (*n* = 198)	≥1	Chronic, persistent and newly ITP	\	6 weeks
Eser (2016) (44)	Multi-center	Eltrombopag (*n* = 31)	Median 4 (3–5)	Chronic ITP	8 (5–16)	Median 29 (11–74) weeks
Gardellini (2021) (45)	Single-center	Eltrombopag (*m* = 18)	≥1	Persistent and chronic ITP	34 (1–76)	Median 21.1 (0.4–64.7) months
Gonzalez-Lopez (2016) (46)	Multi-center	Eltrombopag (*n* = 152)	Median 3 (2–4)	Chronic ITP	22 (8–39)	15 months
Gonzalez-Lopez (2017) (47)	Multi-center	Eltrombopag (*n* = 220)	Median 2 (1,3) (newly), Median 2 (1,2) (persistent), Median 3 (2,4) (chronic)	Newly, persistent and chronic ITP	16 (8, 29) (newly), 14 (7,25) (persistent), 22 (9;38) (chronic)	Median 12 (7,17) months (newly), Median 13 (5, 22) months (persistent), Median 15 (7, 23) months (chronic)
Gonzalez-Lopez (2020) (48)	Multi-center	Eltrombopag (*n* = 106)	Median 2 (2,4)	Newly, persistent and chronic ITP	14 (8,28)	Median 12 (5,19) months
Khellaf (2011) (49)	Multi-center	Romiplostim (*n* = 72)	Median 5 (2–12)	Chronic ITP	11 (1–60)	24 months
Skopec (2021) (55)	Multi-center	Romiplostim (*n* = 100)	≥1	Newly, persistent, chronic ITP	7.5 (4.0, 16.0) (newly), 19.0 (7.0, 45.0) (persistent), 26.0 (14.0, 42.0) (chronic)	24 weeks
Mingot-Castellano (2018) (50)	Multi-center	Eltrombopag and Romiplostim (*n* = 122)	Median 3 (2–4)	Newly, persistent and chronic	11.5 (6–25)	86.5 (34.3–128) weeks
Mishra (2020) (51)	Single-center	Eltrombopag (*n* = 53)	3 (1–8)	Acute, persistent and chronic ITP	10 (1–3)	90 days
Özdemirkıran (2015) (52)	Multi-center	Eltrombopag (*n* = 40)	3 (3–4)	Chronic ITP	Mean 11.5 ± 8.3	Mean 13.78 (7.51) months
Palandri (2021) (53)	Multi-center	Eltrombopag and Romiplostim (*n* = 384)	\	Chronic ITP	20 (1–50)	3 months
Reiser (2022) (54)	Multi-center	Romiplostim (*n* = 96)	≥1	Newly, persistent and chronic ITP	31.5 (21, 50) (newly), 28.0 (19, 78) (persistent), 29.0 (15, 45) (chronic)	24 weeks
Virijević (2022) (56)	Single-center	Eltrombopag and Romiplostim (*n* = 36)	Elt: 4 (3–4), Rom: 5 (4–5)	Chronic ITP	Elt: 11.5 (7–19), Rom: 10 (2–22)	Elt: median 25 (6–36.5) months Rom: median 23.5 (8–37.5) months

All studies were retrospective single-arm studies. Elt, Eltrombopag; ITP, Immune thrombocytopenia; Rom, Romiplostim.

**Figure 2:**
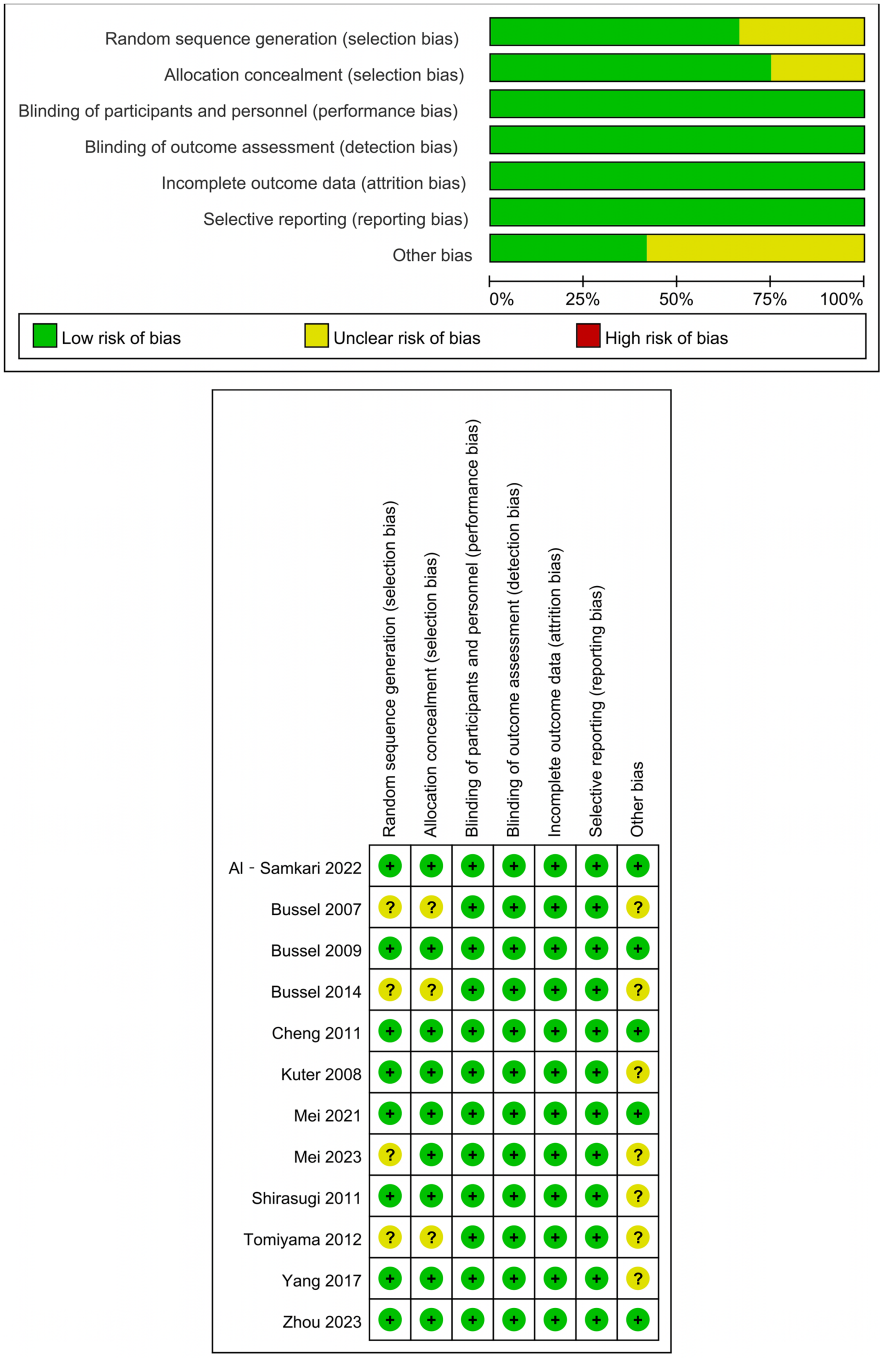
Risk bias of randomized controlled trials.

The methodological rigor assessment revealed low bias risk across all RCTs, detailed in Figure 2. In contrast, observational investigations (prospective and retrospective designs), being single arm in design, exhibited higher risk of bias (Supplementary Table S2).

### 3.2 Efficacy of TPO-RAs treatment

#### 3.2.1 Overall platelet response

**Figure 3:**
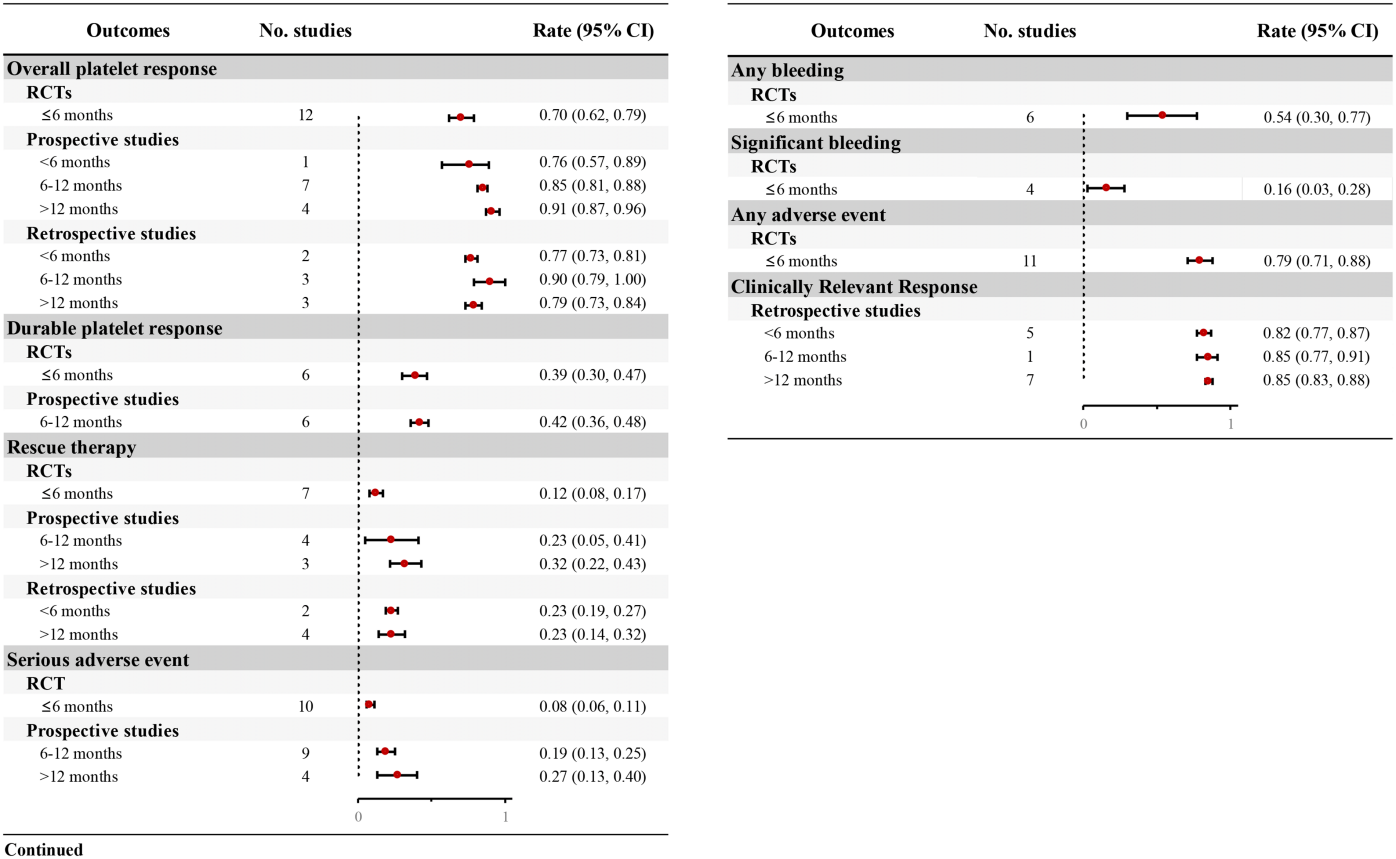
Forest plot of the meta-analysis for major outcomes from RCTs and real-world studies.

Twelve RCTs documented TPO-RA-induced platelet responses (≥50 × 10^9^/L) during short-term therapy (≤6 months). Patients receiving TPO-RAs demonstrated a significantly higher platelet response rate versus placebo (OR = 18.07, 95% CI: 12.4–26.16, *p* < 0.001, *I^2^* = 39.2%), with an overall response rate of 70% (95% CI: 0.62–0.79) (Table 4; Figure 3; Supplementary Table S3). RWS provided additional insights into the overall platelet response for longer-term treatments. One prospective study (38) reported an overall platelet response of 76% (95% CI: 0.57–0.89) within < 6 months of treatment, consistent with RCTs. Seven prospective studies (14, 15, 24, 28, 31, 35, 37) evaluated treatments lasting 6–12 months, demonstrating a response rate of 85% (95% CI: 0.81–0.88). Additionally, four prospective studies (16, 32, 36, 39) reported outcomes for treatments exceeding 12 months, with an overall platelet response rate reaching 91% (95% CI: 0.87–0.96) (Figure 3; Supplementary Table S4). Retrospective studies further corroborated these findings, reporting similar platelet response rates (Supplementary Table S5).

**Table 4:** Results of meta-analysis (randomized controlled trials, main results from trials ≤ 6 months).

Outcomes	Number of studies	Effect model	Results of meta-analysis		Heterogeneity test	
			Odds ratio (95%CI)	*p*	*I^2^*	*p*
Overall platelet response	12 (12–15, 23–30)	Fixed-effect	18.07 (12.48, 26.16)	<0.001	39.2%	0.08
Durable platelet response	6 (12–15, 26, 29)	Fixed-effect	17.48 (8.76, 34.88)	<0.001	0.00%	0.63
Rescue therapy	7 (12–15, 26, 27, 29)	Fixed-effect	0.25 (0.18, 0.35)	<0.001	29.4%	0.20
Any bleeding (WHO1-4)	6 (12, 14, 15, 23, 25, 26)	Fixed-effect	0.43 (0.30, 0.63)	<0.001	0.00%	0.94
Significant bleeding (WHO ≥ 2)	4 (12, 14, 15, 26)	Fixed-effect	0.40 (0.26, 0.61)	<0.001	28.1%	0.24
Any adverse event	11 (12–15, 23, 25–30)	Random-effect	1.38 (0.84, 2.28)	0.20	49.9%	0.03
Serious adverse event	10 (12, 14, 15, 23, 25–30)	Fixed-effect	0.69 (0.47, 1.01)	0.06	23.2%	0.23

Furthermore, most retrospective studies also assessed clinically relevant platelet response, defined as achieving ≥30 × 10^9^/L with ≥100% increase from baseline. Five studies (40, 42, 43, 51, 53) indicated that 82% (95% CI: 0.77–0.87) of patients attained this threshold within 6 month, rising to 85% with prolonged therapeutic exposure (Figure 3; Supplementary Table S5).

#### 3.2.2 Durable platelet response

Six RCTs (12–15, 26, 29) evaluated durable platelet responses during short-term TPO-RAs treatment (≤6 months). Patients receiving TPO-RAs exhibited a significantly higher durable platelet response rate vs. placebo (OR = 17.48, 95% CI: 8.76–34.88, *p* < 0.001, *I^2^* = 0.00%; Table 4), with 39% (95% CI: 0.30–0.47) achieving durable platelet response (Figure 3; Supplementary Table S3). Furthermore, six prospective studies (14, 24, 26, 28, 33, 37) indicated that patients with TPO-RAs were able to achieve durable platelet responses during long-term treatment, with 42% (95% CI: 0.36–0.48) of patients maintaining durable responses over a treatment period of 6–12 months (Figure 3; Supplementary Table S4).

#### 3.2.3 Rescue therapy

Seven RCTs (12–15, 26, 27, 29) quantified requirements of rescue therapy during short-term TPO-RAs administration (≤6 months). TPO-RA recipients exhibited a significantly reduction in rescue therapy necessity compared to placebo (OR = 0.25, 95% CI: 0.18–0.35, *p* < 0.001, *I^2^* = 29.4%; Table 4), with only 12% (95% CI: 0.08–0.17) requiring adjunctive interventions (Figure 3; Supplementary Table S3). RWS provided further insights into the proportion of patients requiring rescue therapy over extended treatment durations. Prospective studies indicated that 23% (95% CI: 0.05–0.41) of patients required rescue therapy at 6–12 months (26, 31, 35, 37), rising to 32% (95% CI: 0.22–0.43) beyond 12 months (16, 36, 39) (Figure 3; Supplementary Table S4). Retrospective studies corroborated these findings, with approximately 23% of patients requiring rescue therapy (Figure 3; Supplementary Table S5).

#### 3.2.4 Bleeding

Six RCTs (12, 14, 15, 23, 25, 26) assessed bleeding incidence using WHO criteria (Grades 1–4) in ITP patients receiving TPO-RAs, while four RCTs (12, 14, 15, 26) reported significant bleeding (Grades ≥ 2). Meta-analysis demonstrated significant lower risk of both any bleeding (OR = 0.43, 95% CI: 0.30–0.63, *p* < 0.001, *I^2^* = 0.00%) and significant bleeding (OR = 0.40, 95% CI: 0.26–0.61, *p* < 0.001, *I^2^* = 28.1%) with TPO-RA therapy versus placebo (Table 4). Heterogeneity in the definitions of bleeding events across observational studies precluded quantitative synthesis of real-world bleeding rates.

### 3.3 Safety of TPO-RAs treatment

#### 3.3.1 Adverse events

Eleven RCTs (12–15, 23, 25–30) evaluated any adverse event profiles during short-term TPO-RAs treatment (≤6 months). The pooled OR indicated comparable any adverse event incidence between TPO-RAs group and placebo group (OR = 1.38, 95% CI: 0.84–2.28, *p* = 0.20, *I^2^* = 49.9%) (Table 4). Similarly, SAEs exhibited non-significant risk differentials (OR = 0.69, 95% CI: 0.47–1.01, *p* = 0.06, *I^2^* = 23.2%), suggesting no statistically meaningful elevation in adverse event liability attributable to TPO-RA therapy.

Thirteen prospective studies demonstrated a progressive increase in SAE incidence compared to RCT benchmarks. While RCTs reported an 8% SAE rate (95% CI: 0.06–0.11), prospective studies increased to 19% (95% CI: 0.13–0.25) at 6–12 month (14, 15, 24, 26, 28, 31, 33–35) and 27% (95% CI: 0.13–0.40) exceeding 12 months (16, 32, 36, 39) (Figure 3; Supplementary Table S4). These findings underscore the necessity for vigilant hematologic and systemic monitoring during chronic TPO-RA therapy to mitigate cumulative toxicity risks.

### 3.4 Publish bias assessments

A funnel plot of primary efficacy outcome was constructed to assess potential publication bias, visual inspection of the funnel plot did not reveal any substantial asymmetry. These findings substantiate the methodological robustness of the meta-analysis, indicating minimal susceptibility to publication bias (Supplementary Figure S1).

## 4 Discussion

The chronic nature of ITP often necessitates long-term treatment with TPO-RAs, making the evaluation of their sustained therapeutic performance important (32). While RCTs establish robust short-term efficacy and safety profiles, lack of long-term follow-up data restricts their ability to inform clinical practice regarding the sustained efficacy and safety of TPO-RAs. To address this critical gap, our meta-analysis innovatively synthesizes RWE with RCT data, revealing two pivotal insights: (1) TPO-RAs demonstrate superior platelet response rates in both short-term (≤6 months) and more long-term treatment periods, and (2) prolonged administration correlates with increased rescue therapy requirement (12 to 32%) and SAE incidence (8 to 27%). These findings highlight the necessity for risk-adapted monitoring protocols that optimize the benefit-to-risk calculus during extended TPO-RA therapy, particularly in patients requiring indefinite thrombopoietic support.

Previous meta-analyses and systematic reviews on TPO-RAs in ITP have predominantly relied on RCT data (18, 57, 58), which are often limited by short follow-up durations and highly controlled patient populations. These limitations leave a critical knowledge gap regarding the long-term durability of platelet responses and the cumulative risk of adverse events, thereby restricting the generalizability of findings to real-world clinical settings. RWS provide valuable insights into the effectiveness and safety of TPO-RAs over extended periods. Unlike previous meta-analyses, this study incorporates both prospective and retrospective RWS to supplement the short-term outcomes reported in RCTs. This approach not only enhances the external validity of our findings but also provides critical insights into the long-term efficacy and safety of TPO-RAs. By bridging the gap between RCTs and RWS, our study offers a more nuanced understanding of TPO-RAs in ITP management, which is essential for guiding clinical decision-making.

This analysis reveals several important findings that have significant implications for clinical practice. First, TPO-RAs demonstrate a high overall platelet response rate in both short-term and long-term treatment periods. In RCTs, the short-term (≤6 months) response rate was 70%, which is consistent with the 76% response rate observed in RWS (38). Notably, the response rate increased to 85% at 6–12 months and reached 91% beyond 12 months. This suggests that TPO-RAs can rapidly induce a high platelet response rate and maintain efficacy over extended periods. However, the increasing need for rescue therapy (23% at 6–12 months and 32% beyond 12 months) highlight the challenges of sustaining long-term responses in some patients. These findings underscore the importance of individualized treatment strategies, regular monitoring, and timely adjustments to therapy to optimize long-term outcomes in ITP management (6).

Second, the meta-analysis confirms that TPO-RAs significantly reduce bleeding risk versus placebo, with lower rates of both any bleeding (OR = 0.43, 95%CI: 0.30–0.63) and significant bleeding (OR = 0.40, 95%CI: 0.26–0.61) during short-term therapy. This is a critical benefit, as bleeding complications are a major cause of morbidity and mortality in ITP patients (28, 58). However, heterogeneity definitions across observational studies precludes quantitative synthesis of real-world bleeding rates, underscoring the imperative for standardized bleeding criteria in longitudinal TPO-RA safety surveillance protocols.

Third, while TPO-RAs were well-tolerated in the short term, with no significant increase in any AEs or SAEs compared to placebo, the risk of SAEs potentially increased with prolonged treatment. The incidence of SAEs rose from 8% in RCTs to 19% at 6–12 months and 27% beyond 12 months in RWS. This trend may reflect the toxicity of long-term TPO-RAs use in real-world populations, emphasizing the importance of regular monitoring and individualized risk–benefit assessments when prescribing TPO-RAs for extended periods.

Beyond these clinical and laboratory endpoints, the impact of TPO-RAs on HRQoL is an important consideration for patients with chronic ITP. A qualitative synthesis of the included RCTs indicates that TPO-RA therapy is associated with meaningful improvements in quality of life. For instance, one study (NCT00102739) reported a significant improvement from baseline in emotional-role scores (*p* = 0.02) for patients receiving eltrombopag (23). Furthermore, another study (NCT00370331) demonstrated that improvements in HRQoL were significantly associated not only with eltrombopag-mediated increases in platelet counts (*p* = 0.034), but also with decreases in WHO bleeding grades (*p* = 0.002) (12). These findings underscore that the benefits of TPO-RAs extend beyond platelet elevation to encompass enhanced overall well-being and daily functioning, which are paramount from the patient’s perspective.

Looking to the future, novel therapeutic modalities such as CAR-T cell therapy have advanced to clinical trials for application in selected patients with treatment-refractory ITP and hold the promise of achieving complete remission or even a cure for ITP (59). Nevertheless, TPO-RAs continue to play an essential role in ITP management due to their well-established efficacy, manageable safety profile, and cost-effectiveness. Consequently, the long-term effectiveness and safety data synthesized in this meta-analysis, which are uniquely derived from RWS, provides critical evidence to inform the sustained and secure use of TPO-RAs in clinical practice.

However, this study has some limitations. First, the heterogeneity in study designs, patient populations, and outcome definitions across RWS posed challenges for data synthesis. Although we used random-effects models to account for this heterogeneity, it may still have influenced our pooled estimates. Second, the lack of long-term data from RCTs limited our ability to directly compare short-term and long-term outcomes. Third, the retrospective nature of some RWS may have introduced bias, particularly in the reporting of adverse event and bleeding events. Fourth, it was not feasible to perform a valid pooled analysis of bleeding events in RWS due to the highly heterogeneous definitions and grading scales employed across the included studies.

Fifth, although a quantitative meta-analysis of HRQoL outcomes was not conducted, we have qualitatively summarized the reported improvements in HRQoL in the Discussion section. Finally, the potential competing risk of death was not addressed in the statistical analysis. However, given that the overall mortality in the included studies was low (see Supplementary Table S6 for details), we believe its impact on the pooled results is likely minimal. Future research should address these limitations by conducting long-term RCTs, standardizing outcome report in RWS.

## 5 Conclusion

In conclusion, this meta-analysis establishes that TPO-RAs are highly effective in achieving and maintaining platelet responses in ITP patients, with significant reductions in bleeding risk and a favorable short-term safety profile. However, the increasing need for rescue therapy and risk of SAEs with prolonged treatment underscores the importance of careful monitoring and individualized treatment strategies. Future research could aim to identify factors influencing durable response and late-emerging adverse events to optimize long-term, personalized patient management.

## Data Availability

The original contributions presented in the study are included in the article/Supplementary material, further inquiries can be directed to the corresponding authors.
