# Supplementary material for: Bridging the gaps between randomized controlled trials and real-world use of thrombopoietin receptor agonists for adult primary immune thrombocytopenia: a systematic review and meta-analysis
Source: Front Med (Lausanne). 2025 Sep 24;12:1667457. doi: 10.3389/fmed.2025.1667457 (PMC12504389; doi:10.3389/fmed.2025.1667457)
Supplement: Supplementary file 1 [file Supplementary_file_1.docx]

***Supplementary Material***

**Supplementary Table S1.** Search strategy for the databases based (PubMed as an example).

| Search number | Query | Results on 23 January 2025 |
| --- | --- | --- |
| #4 | #1 AND #2 AND #3 | 332 |
| #3 | (((((( ("Randomized Controlled Trial" [Publication Type]) OR ("Clinical Trial" [Publication Type])) OR (Real World[Title/Abstract])) OR ("Prospective Studies"[Mesh])) OR ("Retrospective Studies"[Mesh])) OR ("Observational Study" [Publication Type])) OR ("Cohort Studies"[Mesh])) OR ("Case-Control Studies"[Mesh]) | 3,849,069 |
| #2 | (((((((((((("romiplostim" [Supplementary Concept]) OR (Romiplostim[Title/Abstract])) OR (Nplate[Title/Abstract])) OR ("eltrombopag" [Supplementary Concept])) OR (Eltrombopag[Title/Abstract])) OR (Promacta[Title/Abstract])) OR (Revolade[Title/Abstract])) OR ("hetrombopag" [Supplementary Concept])) OR (Hetrombopag[Title/Abstract])) OR ("avatrombopag" [Supplementary Concept])) OR (Avatrombopag[Title/Abstract])) OR (Doptelet[Title/Abstract])) OR (thrombopoietin receptor agonists[Title/Abstract]) | 2,271 |
| #1 | (((((("Purpura, Thrombocytopenic, Idiopathic"[Mesh]) OR (Autoimmune Thrombocytopenia[Title/Abstract])) OR (Immune Thrombocytopenic Purpura[Title/Abstract])) OR (Immune Thrombocytopenia[Title/Abstract])) OR (Werlhof Disease[Title/Abstract])) OR (Autoimmune Thrombocytopenic Purpura[Title/Abstract])) OR (Idiopathic Thrombocytopenic Purpura[Title/Abstract]) | 14,760 |

**Supplementary Table S2.** JBI critical appraisal risk of bias assessment of the case series study.

| **Study** | **[1]** | **[2]** | **[3]** | **[4]** | **[5]** | **[6]** | **[7]** | **[8]** | **[9]** | **[10]** |
| --- | --- | --- | --- | --- | --- | --- | --- | --- | --- | --- |
| **Prospective study** | | | | | | | | | | |
| Janssens (2015) | Y | Y | Y | Y | Y | Y | Y | Y | N | Y |
| Kuter (2013) | Y | N | Y | N | Y | Y | Y | Y | N | Y |
| Newland (2016) | Y | Y | Y | Y | Y | Y | Y | Y | N | Y |
| Wong (2017) | Y | Y | Y | N | Y | Y | Y | Y | N | Y |
| Snell Taylor (2021) | Y | N | Y | Y | Y | Y | Y | Y | N | Y |
| Tripathi (2014) | Y | Y | Y | Y | Y | N | N | Y | N | Y |
| Wong (2023) | Y | N | Y | Y | Y | Y | Y | Y | N | Y |
| Lucchini (2021) | Y | Y | Y | Y | Y | Y | Y | Y | N | Y |
| **Retrospective study** | | | | | | | | | | |
| Arnall (2021) | Y | N | Y | Y | Y | Y | Y | Y | N | Y |
| Çekdemir (2019) | Y | Y | Y | Y | Y | N | N | Y | N | Y |
| Cooper（2024） | Y | N | Y | Y | Y | Y | Y | Y | N | Y |
| Dong (2024) | Y | Y | Y | Y | Y | Y | Y | N | N | Y |
| Eser (2016) | Y | Y | Y | Y | Y | Y | Y | Y | N | Y |
| Gardellini (2021) | Y | Y | Y | Y | Y | Y | Y | Y | N | Y |
| Gonzalez-Lopez (2016) | N | Y | Y | Y | Y | Y | Y | Y | N | Y |
| Gonzalez-Lopez (2017) | N | Y | Y | Y | Y | Y | Y | Y | N | Y |
| Gonzalez-Lopez (2020) | N | Y | Y | Y | Y | Y | Y | N | N | Y |
| Khellaf (2011) | Y | Y | Y | N | Y | Y | Y | N | N | Y |
| Skopec (2021) | Y | N | Y | N | Y | Y | Y | Y | N | Y |
| Mingot-Castellano (2018) | Y | Y | Y | Y | Y | Y | Y | Y | N | Y |
| Mishra (2020) | N | Y | Y | Y | Y | Y | Y | Y | N | Y |
| Özdemirkıran (2015) | Y | Y | Y | Y | Y | Y | Y | Y | N | Y |
| Palandri (2021) | Y | Y | Y | Y | Y | Y | Y | Y | N | Y |
| Reiser (2022) | Y | Y | Y | Y | Y | Y | Y | Y | N | Y |
| Virijević (2022) | N | Y | Y | Y | Y | Y | Y | Y | N | Y |

**Note:** [1] Were there clear criteria for inclusion in the case series? [2] Was the condition measured in a standard, reliable way for all participants included in the case series? [3] Were valid methods used for identification of the condition for all participants included in the case series? [4] Did the case series have consecutive inclusion of participants? [5] Did the case series have complete inclusion of participants? [6] Was there clear reporting of the demographics of the participants in the study? [7] Was there clear reporting of clinical information of the participants? [8] Were the outcomes or follow up results of cases clearly reported? [9] Was there clear reporting of the presenting site(s)/clinic(s) demographic information? [10] Was statistical analysis appropriate? Y: Yes: N: No.

**Supplementary Table S3.** Results of Meta-analysis of Intervention group in randomized controlled trials (main results from trials ≤6 months).

| **Outcomes** | **Number of studies** | **Results of Meta-analysis** | ***I^2^*** | **Effect model** |
| --- | --- | --- | --- | --- |
| **Overall platelet response** | 12(1-12) | 0.70 (0.62, 0.79) | 89.67% | Random-effect |
| **Durable platelet response** | 6(1, 5-8, 11) | 0.39 (0.30, 0.47) | 81.30% | Random-effect |
| **Rescue therapy** | 7(1, 5-9, 11) | 0.12 (0.08, 0.17) | 67.41% | Random-effect |
| **Any bleeding (WHO1-4)** | 6(1, 2, 4, 5, 7, 8) | 0.54 (0.30, 0.77) | 97.95% | Random-effect |
| **Significant bleeding (WHO≥2)** | 4(1, 5, 7, 8) | 0.16 (0.03, 0.28) | 92.50% | Random-effect |
| **Any adverse event** | 11(1, 2, 4-12) | 0.79 (0.71, 0.88) | 93.01% | Random-effect |
| **Serious adverse event** | 10(1, 2, 4, 5, 7-12) | 0.08 (0.06, 0.11) | 58.69% | Random-effect |

**Supplementary Table S4.** Results of Meta-analysis of Prospective studies.

| **Outcomes** | **Number of studies** | **Results of Meta-analysis** | ***I^2^*** | **Effect model** |
| --- | --- | --- | --- | --- |
| **Overall platelet response** |  |  |  |  |
| <6 months | 1(13) | 0.76 (0.57, 0.89) | / | Random-effect |
| 6-12 months | 7(1, 3, 7, 10, 14-16) | 0.85 (0.81, 0.88) | 57.75% | Random-effect |
| >12 months | 4(17-20) | 0.91 (0.87, 0.96) | 82.30% | Random-effect |
| **Durable platelet response (6-12 months)** | 6(3, 7, 8, 10, 16, 21) | 0.42 (0.36, 0.48) | 65.87% | Random-effect |
| **Rescue therapy** |  |  |  |  |
| 6-12 months | 4(8, 14-16) | 0.23 (0.05, 0.41) | 97.51% | Random-effect |
| >12 months | 3(18-20) | 0.32 (0.22, 0.43) | / | Random-effect |
| **Serious adverse event** |  |  |  |  |
| 6-12 months | 9(1, 3, 7, 8, 10, 14, 15, 21, 22) | 0.19 (0.13, 0.25) | 85.51% | Random-effect |
| >12 months | 4(17-20) | 0.27 (0.13, 0.40) | 95.22% | Random-effect |

**Supplementary Table S5.** Results of Meta-analysis of Retrospective studies.

| **Outcomes** | **Number of studies** | **Results of Meta-analysis** | ***I^2^*** | **Effect model** |
| --- | --- | --- | --- | --- |
| **Clinically Relevant Response** |  |  |  |  |
| <6 months | 5(23-27) | 0.82 (0.77, 0.87) | 69.97% | Random-effect |
| 6-12 months | 1(28) | 0.85 (0.77, 0.91) | / | Random-effect |
| >12 months | 7(29-35) | 0.85 (0.83, 0.88) | 2.90% | Random-effect |
| **Overall platelet response** |  |  | 93.88% |  |
| <6 months | 2(24, 25) | 0.77 (0.73, 0.81) | / | Random-effect |
| 6-12 months | 3(28, 36, 37) | 0.90 (0.79, 1.00) | / | Random-effect |
| >12 months | 3(34, 38, 39) | 0.79 (0.73, 0.84) | / | Random-effect |
| **Rescue therapy** |  |  |  |  |
| <6 months | 2(24, 28) | 0.23 (0.19, 0.27) | / | Random-effect |
| >12 months | 4(31, 33, 34, 38) | 0.23 (0.14, 0.32) | 82.17% | Random-effect |

**Supplementary Table S6.** Mortality in TPO-RA vs. Placebo Groups of Randomized Controlled Trials.

| **Author (published year)** | **TPO-RAs group (n, %)** | **Placebo group (n, %)** | **Characteristics of the death patients** |
| --- | --- | --- | --- |
| Al‐Samkari (2022)(1) | 0 | 0 | / |
| Bussel (2007)(2) | 1 (1.1) | 0 | 66 years old, man, who had undergone a pneumonectomy for non–small-cell lung cancer and entered the study with chronic obstructive pulmonary disease, asthma, and peripheral edema. He received 50 mg of eltrombopag for 21 days and had grade 3 pneumonia, hepatitis, and renal insufficiency and grade 4 exacerbation of chronic obstructive pulmonary disease. Twenty-five days after entering the study, the patient died of cardiopulmonary failure. |
| Bussel (2009)(4) | 0 | 0 | / |
| Bussel (2014)(3) | 0 | 0 | / |
| Cheng (2011)(5) | 0 | 1 (1.6) | Fatal brain-stem haemorrhage in placebo group. |
| Kuter (2008)(6) | 0 | 3 (7.1) | Three deaths in the placebo group, but no details were reported. |
| Mei (2021)(7) | 2 (0.6) | 1 (1.2) | NR |
| Mei (2023)(8) | 0 | 0 | / |
| Shirasugi (2011)(9) | 0 | 0 | / |
| Tomiyama (2012)(10) | 0 | 0 | / |
| Yang (2017)(11) | 0 | 1 (1.9) | One death in the placebo group, but no details were reported. |
| Zhou (2023)(12) | 2 (1.3) | 0 | One patient with multiple organ dysfunction syndrome resulted in death; one patient experienced an exacerbation of intracranial hemorrhage due to a low platelet count. |

Abbreviations: NR: Not reported; TPO-RAs: thrombopoietin receptor agonists.


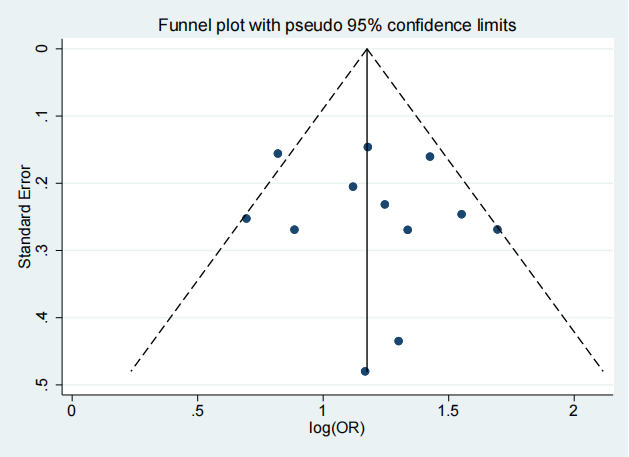


**Supplementary Figure S1.** Funnel plot of the primary efficacy outcome for TOP-RAs treatment of primary ITP.

# **References:**

1. Al‐Samkari H, Nagalla S. Efficacy and Safety Evaluation of Avatrombopag in Immune Thrombocytopenia: Analyses of a Phase Iii Study and Long-Term Extension. *Platelets* (2022) 33(2):257-64. doi: 10.1080/09537104.2021.1881952.

2. Bussel JB, Cheng G, Saleh MN, Psaila B, Kovaleva L, Meddeb B, et al. Eltrombopag for the Treatment of Chronic Idiopathic Thrombocytopenic Purpura. *New England Journal of Medicine* (2007) 357(22):2237-47. doi: 10.1056/NEJMoa073275.

3. Bussel JB, Kuter DJ, Aledort LM, Kessler CM, Cuker A, Pendergrass KB, et al. A Randomized Trial of Avatrombopag, an Investigational Thrombopoietin-Receptor Agonist, in Persistent and Chronic Immune Thrombocytopenia. *Blood* (2014) 123(25):3887-94. Epub 2014/05/08. doi: 10.1182/blood-2013-07-514398.

4. Bussel JB, Provan D, Shamsi T, Cheng G, Psaila B, Kovaleva L, et al. Effect of Eltrombopag on Platelet Counts and Bleeding During Treatment of Chronic Idiopathic Thrombocytopenic Purpura: A Randomised, Double-Blind, Placebo-Controlled Trial. *The Lancet* (2009) 373(9664):641-8. doi: 10.1016/S0140-6736(09)60402-5.

5. Cheng G, Saleh MN, Marcher C, Vasey S, Mayer B, Aivado M, et al. Eltrombopag for Management of Chronic Immune Thrombocytopenia (Raise): A 6-Month, Randomised, Phase 3 Study. *The Lancet* (2011) 377(9763):393-402. doi: 10.1016/S0140-6736(10)60959-2.

6. Kuter DJ, Bussel JB, Lyons RM, Pullarkat V, Gernsheimer TB, Senecal FM, et al. Efficacy of Romiplostim in Patients with Chronic Immune Thrombocytopenic Purpura: A Double-Blind Randomised Controlled Trial. *The Lancet* (2008) 371(9610):395-403. doi: 10.1016/S0140-6736(08)60203-2.

7. Mei H, Liu X, Li Y, Zhou H, Feng Y, Gao G, et al. A Multicenter, Randomized Phase Iii Trial of Hetrombopag: A Novel Thrombopoietin Receptor Agonist for the Treatment of Immune Thrombocytopenia. *Journal of Hematology and Oncology* (2021) 14(1). doi: 10.1186/s13045-021-01047-9.

8. Mei H, Zhou H, Hou M, Sun J, Zhang L, Luo J, et al. Avatrombopag for Adult Chronic Primary Immune Thrombocytopenia: A Randomized Phase 3 Trial in China. *Research and Practice in Thrombosis and Haemostasis* (2023) 7(6). doi: 10.1016/j.rpth.2023.102158.

9. Shirasugi Y, Ando K, Miyazaki K, Tomiyama Y, Okamoto S, Kurokawa M, et al. Romiplostim for the Treatment of Chronic Immune Thrombocytopenia in Adult Japanese Patients: A Double-Blind, Randomized Phase Iii Clinical Trial. *International Journal of Hematology* (2011) 94(1):71-80. doi: 10.1007/s12185-011-0886-8.

10. Tomiyama Y, Miyakawa Y, Okamoto S, Katsutani S, Kimura A, Okoshi Y, et al. A Lower Starting Dose of Eltrombopag Is Efficacious in Japanese Patients with Previously Treated Chronic Immune Thrombocytopenia. *Journal of Thrombosis and Haemostasis* (2012) 10(5):799-806. doi: 10.1111/j.1538-7836.2012.04695.x.

11. Yang R, Li J, Jin J, Huang M, Yu Z, Xu X, et al. Multicentre, Randomised Phase Iii Study of the Efficacy and Safety of Eltrombopag in Chinese Patients with Chronic Immune Thrombocytopenia. *British Journal of Haematology* (2017) 176(1):101-10. doi: 10.1111/bjh.14380.

12. Zhou H, Zhou J, Wu D, Ma L, Du X, Niu T, et al. Romiplostim in Primary Immune Thrombocytopenia That Is Persistent or Chronic: Phase Iii Multicenter, Randomized, Placebo-Controlled Clinical Trial in China. *Research and Practice in Thrombosis and Haemostasis* (2023) 7(5). doi: 10.1016/j.rpth.2023.100192.

13. Tripathi AK, Shukla A, Mishra S, Yadav YS, Yadav DK. Eltrombopag Therapy in Newly Diagnosed Steroid Non-Responsive Itp Patients. *International Journal of Hematology* (2014) 99(4):413-7. doi: 10.1007/s12185-014-1533-y.

14. Janssens A, Tarantino M, Bird RJ, Mazzucconi MG, Boccia RV, Fernández MF, et al. Romiplostim Treatment in Adults with Immune Thrombocytopenia of Varying Duration and Severity. *Acta Haematol* (2015) 134(4):215-28. Epub 2015/06/13. doi: 10.1159/000381657.

15. Newland A, Godeau B, Priego V, Viallard JF, López Fernández MF, Orejudos A, et al. Remission and Platelet Responses with Romiplostim in Primary Immune Thrombocytopenia: Final Results from a Phase 2 Study. *Br J Haematol* (2016) 172(2):262-73. Epub 2015/11/06. doi: 10.1111/bjh.13827.

16. Snell Taylor SJ, Nielson CM, Breskin A, Saul B, Yu Y, Alam N, et al. Effectiveness and Safety of Romiplostim among Patients with Newly Diagnosed, Persistent and Chronic Immune Thrombocytopenia in European Clinical Practice. *Advances in Therapy* (2021) 38(5):2673-88. doi: 10.1007/s12325-021-01727-5.

17. Kuter DJ, Bussel JB, Newland A, Baker RI, Lyons RM, Wasser J, et al. Long-Term Treatment with Romiplostim in Patients with Chronic Immune Thrombocytopenia: Safety and Efficacy. *Br J Haematol* (2013) 161(3):411-23. Epub 2013/02/26. doi: 10.1111/bjh.12260.

18. Shirasugi Y, Ando K, Miyazaki K, Tomiyama Y, Iwato K, Okamoto S, et al. An Open-Label Extension Study Evaluating the Safety and Efficacy of Romiplostim for up to 3.5 Years in Thrombocytopenic Japanese Patients with Immune Thrombocytopenic Purpura (Itp). *Int J Hematol* (2012) 95(6):652-9. Epub 2012/04/26. doi: 10.1007/s12185-012-1065-2.

19. Wong RSM, Saleh MN, Khelif A, Salama A, Portella MSO, Burgess P, et al. Safety and Efficacy of Long-Term Treatment of Chronic/Persistent Itp with Eltrombopag: Final Results of the Extend Study. *Blood* (2017) 130(23):2527-36. Epub 2017/10/19. doi: 10.1182/blood-2017-04-748707.

20. Wong RSM, Yavaşoğlu İ, Yassin MA, Tarkun P, Yoon SS, Wei X, et al. Eltrombopag in Patients with Chronic Immune Thrombocytopenia in Asia-Pacific, the Middle East, and Turkey: Final Analysis of Cite. *Blood Adv* (2023) 7(17):4773-81. Epub 2022/09/15. doi: 10.1182/bloodadvances.2022008287.

21. Liu X, Hou M, Li J, Jin J, Huang M, Yu Z, et al. Efficacy and Safety of Eltrombopag in Chinese Patients with Chronic Immune Thrombocytopenia: Stage 2 Results from a Multicenter Phase Iii Study. *Platelets* (2022) 33(1):82-8. doi: 10.1080/09537104.2020.1847267.

22. Lucchini E, Palandri F, Volpetti S, Vianelli N, Auteri G, Rossi E, et al. Eltrombopag Second-Line Therapy in Adult Patients with Primary Immune Thrombocytopenia in an Attempt to Achieve Sustained Remission Off-Treatment: Results of a Phase Ii, Multicentre, Prospective Study. *British Journal of Haematology* (2021) 193(2):386-96. doi: 10.1111/bjh.17334.

23. Arnall JR, DiSogra KY, Downing L, Elmes JB, Tran T, Moore DC. Comparative Utilization and Efficacy of Thrombopoietin Receptor Agonists in Relapsed/Refractory Immune Thrombocytopenia. *Am J Ther* (2021) 28(5):e525-e30. Epub 2021/01/26. doi: 10.1097/mjt.0000000000001335.

24. Cooper N, Scully M, Percy C, Nicolson PLR, Lowe G, Bagot CN, et al. Real-World Use of Thrombopoietin Receptor Agonists for the Management of Immune Thrombocytopenia in Adult Patients in the United Kingdom: Results from the Trait Study. *British Journal of Haematology* (2024) 204(6):2442-52. doi: 10.1111/bjh.19345.

25. Dong XF, Li YL, Li NB, Lin WN, Wang T, Wang HQ, et al. [Efficacy and Safety of Eltrombopag in the Treatment of Primary Immune Thrombocytopenia: Real-World Data from a Single Medical Center]. *Zhonghua Xue Ye Xue Za Zhi* (2024) 45(3):271-6. Epub 2024/05/08. doi: 10.3760/cma.j.cn121090-20231108-00257.

26. Mishra K, Pramanik S, Jandial A, Sahu KK, Sandal R, Ahuja A, et al. Real-World Experience of Eltrombopag in Immune Thrombocytopenia. *American Journal of Blood Research* (2020) 10(5):240-51.

27. Palandri F, Rossi E, Bartoletti D, Ferretti A, Ruggeri M, Lucchini E, et al. Real-World Use of Thrombopoietin Receptor Agonists in Older Patients with Primary Immune Thrombocytopenia. *Blood* (2021) 138(7):571-83. doi: 10.1182/blood.2021010735.

28. Skopec B, Sninska Z, Tzvetkov N, Ivanushkin V, Björklöf K, Hippenmeyer J, et al. Effectiveness and Safety of Romiplostim among Patients with Newly Diagnosed, Persistent and Chronic Itp in Routine Clinical Practice in Central and Eastern Europe: An Analysis of the Platon Study. *Hematology (United Kingdom)* (2021) 26(1):497-502. doi: 10.1080/16078454.2021.1948209.

29. Çekdemir D, Güvenç S, Özdemirkıran F, Eser A, Toptaş T, Özkocaman V, et al. A Multi-Center Study on the Efficacy of Eltrombopag in Management of Refractory Chronic Immune Thrombocytopenia: A Real-Life Experience. *Turkish Journal of Hematology* (2019) 36(4):230-7. doi: 10.4274/tjh.galenos.2019.2018.0307.

30. Gardellini A, Guidotti F, Feltri M, Zancanella M, Maino E, Ambrosiani L, et al. Eltrombopag as Second Line Treatment in Patients with Primary Immune Thrombocytopenia: A Single Center Real Life Experience. *Blood Cells, Molecules, and Diseases* (2021) 92. doi: 10.1016/j.bcmd.2021.102620.

31. González-López TJ, Alvarez-Román MT, Pascual C, Sánchez-González B, Fernández-Fuentes F, Jarque I, et al. Eltrombopag Safety and Efficacy for Primary Chronic Immune Thrombocytopenia in Clinical Practice. *European Journal of Haematology* (2016) 97(3):297-302. doi: 10.1111/ejh.12725.

32. González-López TJ, Fernández-Fuertes F, Hernández-Rivas JA, Sánchez-González B, Martínez-Robles V, Alvarez-Román MT, et al. Efficacy and Safety of Eltrombopag in Persistent and Newly Diagnosed Itp in Clinical Practice. *International Journal of Hematology* (2017) 106(4):508-16. doi: 10.1007/s12185-017-2275-4.

33. González-López TJ, Sánchez-González B, Jarque I, Bernat S, Fernández-Fuertes F, Caparrós I, et al. Use of Eltrombopag for Patients 65 years Old or Older with Immune Thrombocytopenia. *European Journal of Haematology* (2020) 104(3):259-70. doi: 10.1111/ejh.13370.

34. Mingot-Castellano ME, Caparrós IS, Fernández F, Perera-Alvarez MDM, Jimenez-Bárcenas R, Casaus García A, et al. Treatment Characteristics, Efficacy and Safety of Thrombopoietin Analogues in Routine Management of Primary Immune Thrombocytopenia. *Blood coagulation & fibrinolysis : an international journal in haemostasis and thrombosis* (2018) 29(4):374-80. Epub 2018/05/09. doi: 10.1097/mbc.0000000000000726.

35. Özdemirkıran F, Payzın B, Kiper HD, Kabukçu S, Çağlıyan GA, Kahraman S, et al. Eltrombopag for the Treatment of Immune Thrombocytopenia: The Aegean Region of Turkey Experience. *Turkish Journal of Hematology* (2015) 32(4):323-8. doi: 10.4274/tjh.2014.0152.

36. Eser A, Toptas T, Kara O, Sezgin A, Noyan-Atalay F, Yilmaz G, et al. Efficacy and Safety of Eltrombopag in Treatment-Refractory Primary Immune Thrombocytopenia: A Retrospective Study. *Blood Coagulation & Fibrinolysis* (2016) 27(1):47-52. doi: 10.1097/mbc.0000000000000380.

37. Reiser M, Josten KM, Dietzfelbinger H, Seesaghur A, Schill M, Hippenmeyer J, et al. Romiplostim for Primary Immune Thrombocytopenia in Routine Clinical Practice: Results from a Multicentre Observational Study in Germany. *Acta Haematologica* (2022) 145(4):394-403. doi: 10.1159/000521689.

38. Khellaf M, Michel M, Quittet P, Viallard JF, Alexis M, Roudot-Thoraval F, et al. Romiplostim Safety and Efficacy for Immune Thrombocytopenia in Clinical Practice: 2-Year Results of 72 Adults in a Romiplostim Compassionate-Use Program. *Blood* (2011) 118(16):4338-45. Epub 2011/08/13. doi: 10.1182/blood-2011-03-340166.

39. Virijević M, Mitrović M, Pantić N, Pravdić Z, Sabljić N, Suvajdžić-Vuković N. The Role of Thrombopoietin Receptor Agonists in the Management of Adult Primary Immune Thrombocytopenia – a Single Center Experience. *Vojnosaniti Pregl* (2022) 79(10):958-62. doi: 10.2298/VSP210721090V.
